# Metformin improves the angiogenic potential of human CD34^+^ cells co-incident with downregulating *CXCL10* and *TIMP1* gene expression and increasing VEGFA under hyperglycemia and hypoxia within a therapeutic window for myocardial infarction

**DOI:** 10.1186/s12933-016-0344-2

**Published:** 2016-02-09

**Authors:** Sherin Bakhashab, Fahad W. Ahmed, Hans-Juergen Schulten, Ayat Bashir, Sajjad Karim, Abdulrahman L. Al-Malki, Mamdooh A. Gari, Adel M. Abuzenadah, Adeel G. Chaudhary, Mohammed H. Alqahtani, Sahira Lary, Farid Ahmed, Jolanta U. Weaver

**Affiliations:** Institute of Cellular Medicine, Newcastle University, Framlington Place, Newcastle upon Tyne, NE2 4HH UK; Queen Elizabeth Hospital, Gateshead, Newcastle upon Tyne, UK; Biochemistry Department, King Abdulaziz University, Jeddah, Saudi Arabia; Center of Excellence in Genomic Medicine Research, King Abdulaziz University, Jeddah, Saudi Arabia

**Keywords:** Hypoxia, Hyperglycemia, Angiogenesis, CD34^+^ stem cells, Metformin

## Abstract

**Background:**

Cardiovascular disease (CVD) is the leading cause of morbidity and mortality in patients with diabetes mellitus (DM). To identify the most effective treatment for CVD, it is paramount to understand the mechanism behind cardioprotective therapies. Although metformin has been shown to reduce CVD in Type-2 DM clinical trials, the underlying mechanism remains unexplored. CD34^+^ cell-based therapies offer a new treatment approach to CVD. The aim of this study was to investigate the effect of metformin on the angiogenic properties of CD34^+^ cells under conditions mimicking acute myocardial infarction in diabetes.

**Methods:**

CD34^+^ cells were cultured in 5.5 or 16.5 mmol/L glucose ± 0.01 mmol/L metformin and then additionally ± 4 % hypoxia. The paracrine function of CD34^+^ cell-derived conditioned medium was assessed by measuring pro-inflammatory cytokines, vascular endothelial growth factor A (VEGFA), and using an in vitro tube formation assay for angiogenesis. Also, mRNA of CD34^+^ cells was assayed by microarray and genes of interest were validated by qRT-PCR.

**Results:**

Metformin increased in vitro angiogenesis under hyperglycemia–hypoxia and augmented the expression of VEGFA. It also reduced the angiogenic-inhibitors, chemokine (C–X–C motif) ligand 10 (*CXCL10*) and tissue inhibitor of metalloproteinase 1 (*TIMP1*) mRNAs, which were upregulated under hyperglycemia–hypoxia. In addition metformin, increased expression of STEAP family member 4 (*STEAP4*) under euglycemia, indicating an anti-inflammatory effect.

**Conclusions:**

Metformin has a dual effect by simultaneously increasing VEGFA and reducing *CXCL10* and *TIMP1* in CD34^+^ cells in a model of the diabetic state combined with hypoxia. Therefore, these angiogenic inhibitors are promising therapeutic targets for CVD in diabetic patients. Moreover, our data are commensurate with a vascular protective effect of metformin and add to the understanding of underlying mechanisms.

**Electronic supplementary material:**

The online version of this article (doi:10.1186/s12933-016-0344-2) contains supplementary material, which is available to authorized users.

## Background

Cardiovascular disease (CVD) remains the major cause of morbidity and mortality worldwide [1]. The increased risk of CVD in patients with diabetes is well established, and their disease progression is greater than for non-diabetic patients despite similar interventions [2]. CVD is the major cause of mortality in diabetic patients accounting for more than 50 % of all fatalities [2]. Furthermore, the outcome of CVD interventions in patients with diabetes is much worse in comparison with non-diabetic individuals [3, 4]. Impaired angiogenesis is an important factor leading to the development of diabetes associated CVD [5, 6]. Thus, there is an increasing demand for an investigation of new therapeutic targets and treatment approaches directed at restoring angiogenesis in diabetic CVD cases.

CD34^+^ cells alone or as part of bone marrow aspirate have been used as novel stem cell therapies in the treatment of acute myocardial infarction (MI) or heart failure with beneficial effect [7]. A study in SCID mice after experimental MI showed that the primary mode of action of CD34^+^ cells is paracrine [8]. Paracrine secretion and tissue repair were reported to be impaired in CD34^+^ cells from diabetic patients in comparison to non-diabetic or healthy volunteers [9–11]. Patients with diabetes have increased circulating levels of inflammatory markers including tumor necrosis factor-α (TNF-α), interleukin-6 (IL-6), and chemokine (C–C motif) ligand 2 (CCL2) [12]. In addition, increased levels of inflammatory markers predict cardiovascular risk in diabetic patients [13].

Metformin is the anti-diabetic drug that reduced CVD in Type-2 diabetes mellitus (DM) clinical trial (UKPDS) [14]. Although clinical studies provide plausible evidence that metformin improves endothelial function [15–17], the underlying mechanism is not clearly understood. In the current proof of concept study, we used human CD34^+^ cells to understand the beneficial effect of metformin on angiogenesis at 3 h of hypoxia, as this is a therapeutic window in the management of acute myocardial infarction. As umbilical cord blood (UCB) is a non-invasive source of CD34^+^ cells with a higher number of primitive CD34^+^ cells than bone marrow we used UCB derived CD34^+^ cells in our experiments [18, 19].

We reasoned that understanding the cardioprotective mechanism behind metformin therapy will underpin the identification of potential new therapeutic targets and treatment approaches to CVD, particularly in diabetic patients.

## Methods

### Tissue supply

Human umbilical cord blood was collected in 250 mL blood collection bags (Macropharma, Tourcoing, France) for the isolation of CD34^+^ cells. Human umbilical cords were collected and preserved in 50 mL conservation buffer containing Dulbecco’s phosphate buffer saline without Ca^+2^ and Mg^+2^, 200 U/mL penicillin, 200 µg/mL streptomycin, and 2.5 µg/mL fungizone (Gibco, Life Technologies, Paisley, UK).

The study was approved by NHS Health Research Authority, NRES Committee North East-Sunderland, UK (12/NE/0044) and subjects gave informed written consent.

### CD34^+^ cell culture

CD34^+^ cells were isolated from three different UCB collections per condition (10 conditions studied) using Lymph prep protocol and CD34 Microbeads separation according to the manufacturer’s instructions (Miltenyi Biotic Inc., Bergisch Gladbach, Germany). The purity of isolated CD34^+^ cells was assessed by flow cytometry (FACSCanto II, BD, Bioscience, San Jose, CA). All samples showed purity >95 %.

Approximately 1 × 10^6^ CD34^+^ cells per condition were cultured in M199 medium supplemented with 2.0 mmol/L l-glutamate, 100 Units/mL penicillin, 100 μg/mL streptomycin, 2.5 µg/mL fungizone (Gibco, Life Technologies, Paisley, UK), 0.25 % (v/v) human serum albumin (Octapharma, Manchester, UK), 15 mM HEPES, 1.35 g/L NaHCO_3_ (GE Healthcare, Little Chalfont, UK), 100 ng/mL Flt3-L, and 100 ng/mL stem cell factor (PerproTech, Rocky Hill, NJ, USA).

CD34^+^ cells were incubated in a culture medium with 5.5 mmol/L (euglycemia) or 16.5 mmol/L (hyperglycemia) glucose concentrations (Sigma-Aldrich, Dorset, UK). Glucose at 16.5 mmol/L simulates a diabetic state but did not impair cellular growth, whereas increased the expression of endothelial adhesion molecules, consistent with hyperglycemia-mediated vascular inflammation [20–22]. The cells were simultaneously incubated in the presence or absence of 0.01 mmol/L metformin (Sigma-Aldrich, Dorset, UK) for 24 h. The metformin concentration used was selected according to the peak plasma concentration and metformin pharmacokinetics reported earlier in diabetic patients [23]. Subsequently, cells were subjected additionally to 4 % hypoxia (Heracell™ 150i, ThermoScientific) for 3 h to simulate cellular and molecular responses following ischemia [24].

### Meso scale discovery (MSD) assay

The culture medium was collected from 5 × 10^5^ CD34^+^ cells exposed to the above mentioned conditions and assayed using K15025C human Pro-inflammatory II 4-Plex, K151A0H Custom V-PLEX Human Biomarkers (Meso Scale Discovery, Rockville, MD) in accordance with the manufacturer protocol. Plates were read with MSD Sector Imager 2400 and data were analyzed by MSD Discovery Workbench version 2.0 software. The data were normalized against the complete culture medium, and three samples were processed in duplicates.

### HUVEC cultures

HUVEC were harvested from three independent umbilical cords as previously described by us [21]. All experiments were performed at passage two and 80 % confluency.

### In vitro Matrigel tube formation assay

HUVEC (2.0 × 10^4^ cells) were serum starved overnight in M199 complete medium with 0.25 % FBS. An in vitro tube formation assay was performed as previously described with minor modifications [25]. EBM-2 medium containing 14 µmol/L sunitinib malate (VEGFA inhibitor, Sigma-Aldrich, Dorset, UK) was used as a negative control, and this concentration showed no toxicity in HUVEC cell cultures as illustrated in Additional file 1: Figure S1. Subsequently, the cells were incubated at 37 °C and 5 % CO_2_ (OkoLab, NA, Italy) for 24 h in a chamber that was connected to a camera (Hamamatsu Orca ER, Hamamatsu City, Japan). Tube formation was examined by phase-contrast microscopy (Nikon Eclipse Tie, Tokyo, Japan) using software based autofocus (NIS Elements V4.0, Nikon, Tokyo, Japan). Images were acquired every hour. All conditions in each experiment were assessed in duplicate, and tube length was measured as the mean summed length of capillary-like structures in 2 wells. Three independent experiments were performed for each condition. The tube length was measured using Adobe Acrobat Professional version 8 software by measuring long tubes first and then the small branches to cover the whole image. The analysis was conducted blinded.

### Total RNA extraction

Total RNA from CD34^+^ cells was extracted using the RNeasy Mini Kit (QIAGEN, Hilden, Germany) according to the manufacturer’s instructions. The cell lysates from three different CD34^+^ cultures per condition were pooled before proceeding to an RNeasy Mini spin column. On-column DNase digestion with RNase-Free DNase set (QIAGEN) was performed. The quality and quantity of RNA was assessed using a NanoDrop 2000c Spectrophotometer (Thermo Scientific, Wilmington, DE, USA). The integrity of RNA samples was assessed by using Agilent 2100 Bioanalyzer (Santa Clara, CA, USA) yielding high RNA Integrity Numbers (RIN) between 7.0 and 9.7.

### Microarray experiments and gene expression analysis

Microarray experiments were performed using Affymetrix (Santa Clara, CA, USA) Human Gene 1.0 ST arrays according to manufacturer’s instructions with minor modifications [21, 26]. Two technical replicates were hybridized for each experimental condition resulting in a total of 20 microarray experiments.

Affymetrix CEL files were imported to Partek Genomic Suite version 6.6 (Partek Inc., MO, USA). The data were normalized using Robust Multichip Average (RMA) normalization. Principal component analysis (PCA) was performed on all probes to visualize high-dimensional data. By default, expression values were filtered for statistical significance using Benjamini and Hochberg’s False Discovery Rate (FDR), with an FDR-unadjusted *p* value <0.05. ANOVA was performed using the commonly employed *p* values <0.05 and cut off fold change (FC) ≥1.5 as described by Peart et al. [27] and Raouf et al. [28].

Two-dimensional average linkage hierarchical clustering was performed for the differentially expressed genes using Spearman’s correlation as a similarity matrix. The microarray data generated in this study are in compliance with MIAME (http://www.mged.org/Workgroups/MIAME/miame.html) guidelines. The complete dataset and associated experimental information were submitted to NCBI’s Gene Expression Omnibus (GEO) and were accessible through accession number GSE46262. Ingenuity pathway analysis (IPA) software version 9 (Ingenuity, Redwood City, CA, USA) was employed to enable exploring the Canonical Pathways that may be increased or decreased based on activation or inhibition of molecules within that pathway. Additionally, IPA assisted in detecting the interactive molecular and cellular functions affected in each condition.

### Quantitative RT-PCR

Approximately 100 ng of total RNA from each sample was converted to cDNA using SuperScript VILO™ cDNA Synthesis Kit (Life Technologies, Paisley, UK) in a final volume of 20 μL. The cDNA product was quantified by hydrolysis probe real-time PCR performed with TaqMan^®^ Universal Master Mix II and assayed on a 7900HT Fast Real-time PCR system (Life Technologies) according to the manufacturer’s recommended conditions. Expression of the following genes were determined using the TaqMan gene expression assays (Life Technologies): *CCL2*: Hs00234140_m1, *CCL5*: Hs00174575_m1, C–X–C motif chemokine 10 (*CXCL10*): Hs01124251_g1, hepatocyte growth factor (*HGF*): Hs00900070_m1, *IL*-*1α*: Hs00174092_m1, *IL*-*6*: Hs00985639_m1, *IL*-*8*: Hs00174103_m1, tissue metallopeptidase inhibitor 1 (*TIMP1*): Hs00171558_m1, selectin P (*SELP*): Hs00927900_m1. All samples were run in triplicate, and average values were calculated. Three independent reverse transcriptions were tested for each gene. The Comparative C_t_ method (ΔΔCt) was used to quantify expression of the target genes, which were normalized to the endogenous control gene for the large ribosomal protein P0 (*RPLP0*) [21] (Catalog number 432631, Life Technologies).

### Statistical analysis

Results are presented as mean ± SEM, and statistical analysis was performed using one-way ANOVA followed by post hoc analysis using Fisher’s least significant difference (LSD) test for the in vitro Matrigel tube formation assay, qRT-PCR, and MSD assay. Calculations were performed using IBM SPSS software version 21.0 (SPSS Inc, NY). A *p* value <0.05 was considered statistically significant.

## Results

### Secretion of pro-inflammatory and pro-angiogenic cytokines by CD34^+^ cells

**Fig. 1:**
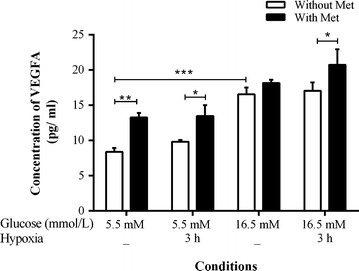
Expression of pro-angiogenic factor VEGFA in CD34^+^ cell-derived conditioned media. The levels of VEGFA were assayed in three independent biological replicates using the MSD technique. Results are presented as ±SEM and were statistically analyzed using one-way ANOVA followed by Fisher’s LSD test. Data for effects of hypoxia and hyperglycemia were compared with control (5.5 mmol/L), and data for cells treated with metformin were compared with the corresponding metformin-untreated condition. *P < 0.05, **P < 0.01, ***P < 0.001

Measured concentrations of the pro-inflammatory cytokines IL-1β, IL-6, IL-8, and TNF-α; were very low in conditioned medium (CM) of CD34^+^ cells under all conditions studied. However, the pro-angiogenic factor VEGFA was found to be significantly increased in CM collected from CD34^+^ cells treated with hyperglycemia (2.0-fold, *p* < 0.001) versus euglycemia, whereas no change was observed under hyperglycemia combined with hypoxia for 3 h versus hyperglycemia. The CM from CD34^+^ cells treated with metformin displayed augmented levels of VEGFA either under euglycemia (1.6-fold, *p* = 0.008), euglycemia combined with hypoxia (1.4-fold, *p* = 0.037), and hyperglycemia combined with hypoxia (1.2-fold, *p* = 0.037) compared with the metformin-untreated condition (Fig. 1).

### The effect of metformin on in vitro angiogenic function of CD34^+^ cell-derived CM

**Fig. 2:**
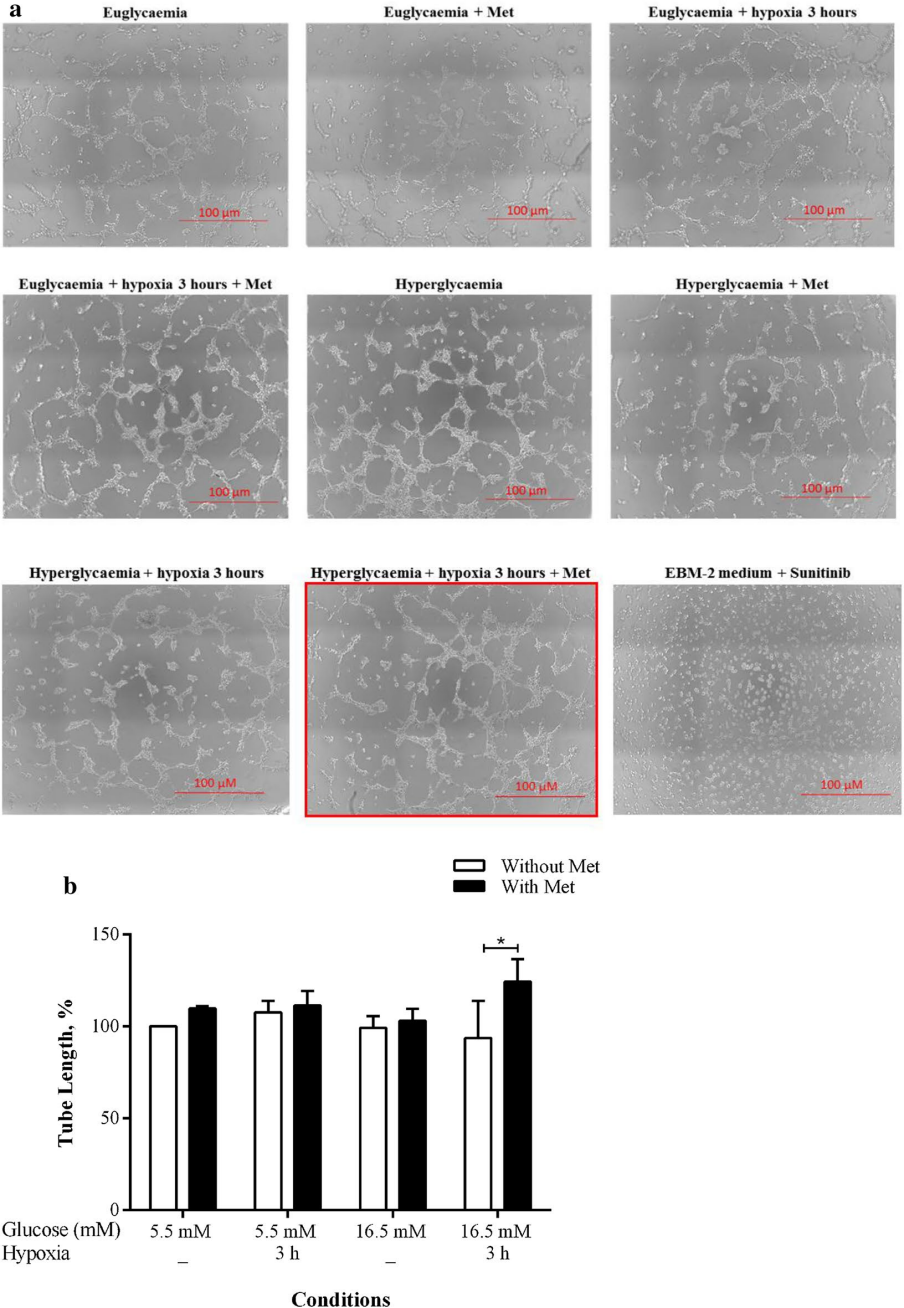
Effect of CM from CD34^+^ cells exposed to different conditions on in vitro angiogenic assays. **a** HUVEC were plated on Matrigel with CM from 2.0 × 10^5^ CD34^+^ cells treated with euglycemia, euglycemia and hypoxia, hyperglycemia or hyperglycemia and hypoxia in the presence and absence of metformin. EBM-2 medium containing the VEGF inhibitor sunitinib (14 µmol/L) was used as a negative control for the assay. The *highlighted image* shows the greatest tube length, which was achieved in HUVEC incubated with CM-derived from CD34^+^ cells treated with hyperglycemia and hypoxia in the presence of metformin. **b** Tube length at 6 h was expressed as a percentage of the tube length of HUVEC treated with 5.5 mmol/L glucose CM (n = 3); *P < 0.05 compared pairwise, i.e., condition with metformin versus metformin-untreated condition

Metformin significantly increased tube formation by 30.7 % (*p* = 0.04) in HUVEC incubated with CD34^+^ CM derived from cells treated with hyperglycemia–hypoxia compared with the condition without metformin (Fig. 2a highlighted with red and b). However, under other conditions metformin did not cause any significant increase in tube formation.

### Metformin drives a gene expression profile consistent with an anti-inflammatory action under euglycemia and euglycemia-hypoxia

The transcriptomic analysis of CD34^+^ cells showed that 313 genes passed the cutoff FC of 1.5 and *p* value <0.05 under euglycemia and metformin compared to euglycemia alone. The top associated molecular and cellular functions predicted using IPA were inflammatory response (*p* = 1.52E−06) and binding of EC (*p* = 1.56E−06), which were inhibited by metformin. The corresponding downregulated genes are highlighted in Table 1.

**Table 1:** Anti-inflammatory genes affected by metformin in CD34^+^ cells under euglycemia identified by IPA

Gene name	Gene symbol	FC	Family
Asp (abnormal spindle) homolog, microcephaly associated (Drosophila)	ASPM	−1.52	Other
Complement component 3	C3	−1.51	Peptidase
Chemokine (C–C motif) ligand 2	CCL2	−1.68	Cytokine
Chemokine (C–C motif) ligand 5	CCL5	−1.81	Cytokine
CD24 molecule	CD24	−1.61	Other
Centromere protein E, 312 kDa	CENPE	−1.51	Other
C-type lectin domain family 2, member D	CLEC2D	−1.59	Transmembrane receptor
Chymase 1, mast cell	CMA1	1.58	Peptidase
Cathepsin L1	CTSL1	−1.50	Peptidase
Glomulin, FKBP associated protein	GLMN	−1.62	Other
Heparanase	HPSE	−1.60	Enzyme
Immunoglobulin heavy constant epsilon	IGHE	1.57	Other
Interleukin 18 receptor 1	IL-18R1	−1.56	Transmembrane receptor
Interleukin 1, alpha	IL-1α	−3.66	Cytokine
Interleukin 26	IL-26	1.53	Cytokine
Interleukin 36, gamma	IL-1F9	−1.56	Cytokine
Interleukin 6 (interferon, beta 2)^a^	IL-6	−2.21	Cytokine
Interleukin 8^a^	IL-8	−1.55	Cytokine
Integrin, beta 3 (platelet glycoprotein IIIa, antigen CD61)	ITGB3	−1.51	Transmembrane receptor
Met proto-oncogene (hepatocyte growth factor receptor)	MET	−1.63	Kinase
Antigen identified by monoclonal antibody Ki-67	MKI67	−1.61	Other
Nuclear receptor corepressor 2	NCOR2	1.77	Transcription regulator
Pro-platelet basic protein (chemokine (C–X–C motif) ligand 7)	PPBP	−1.69	Cytokine
S100 calcium binding protein A8	S100A8	1.71	Other
Serpin peptidase inhibitor, clade B (ovalbumin), member 2	SERPINB2	−4.62	Other
Sclerostin	SOST	1.56	Other
SPC25, NDC80 kinetochore complex component, homolog (*S. cerevisiae*)	SPC25	−1.50	Other
STEAP family member 4	STEAP4	1.90	Oxidoreductase
Thrombospondin 1	THBS1	−1.50	Other
Tumour necrosis factor, alpha-induced protein 6^a^	TNFAIP6	−2.36	Other

The gene list was created by importing Affymetrix .CEL files to Partek Genomic Suite version 6.6. The data were Robust Multichip Average (RMA) normalized. The list of differentially expressed genes was generated using one-way ANOVA, applying FDR-unadjusted *p* value <0.05 with a fold change cutoff of 1.5. Then the list of anti-inflammatory genes was created by IPA software according to the associated molecular and cellular functions

^a^Genes assessed by cytokine assays for paracrine secretion

One hundred genes were differentially expressed in CD34^+^ cells (cutoff FC of 1.5 and *p* value <0.05) under euglycemia and hypoxia compared to euglycemia alone. The top molecular and cellular functions affected as predicted by IPA are reported in Additional file 1: Table S1. Metallothionein 2A (MT2A) was upregulated (1.7-fold, *p* = 4.70E−03) under hypoxia which was previously indicated as one of hypoxia inducible genes [29].

Metformin treatment of CD34^+^ cells exposed to euglycemia and hypoxia caused differential expression of 21 genes compared with the metformin-untreated condition. The top related molecular functions predicted by IPA were: cell death/survival (*p* = 7.23E−04), and cellular growth/proliferation (*p* = 7.23E−04). Genes associated with these functions were *IL*-*5* (1.52-fold, *p* = 7.05E−03), serine peptidase inhibitor, Kazal type 7 (putative) (*SPINK7*, 1.60-fold, *p* = 1.48E−02), and transducer of ERBB2, 2 (*TOB2*, −1.53-fold, *p* = 4.62E−02).

Metformin had no effect on inflammatory response, cell movement, or adhesion under euglycemia-hypoxia as demonstrated in Additional file 1: Figure S2.

### Metformin drives a gene expression profile consistent with improved cell survival in CD34^+^ cells under hyperglycemia

Analysis of microarray data revealed that 370 genes passed the cutoff FC of 1.5 and *p* value <0.05 under hyperglycemia versus euglycemia. Approximately 35 % of the genes were upregulated, and 65 % were downregulated. The uppermost 30 differentially expressed genes are listed in Additional file 1: Table S2. The top molecular functions predicated by IPA were cell to cell signaling and interaction (p = 1.80E−17), cellular growth/proliferation (*p* = 1.21E−12), inflammatory response (*p* = 1.59E−12), cellular movement (*p* = 2.60E−11) which were all predicted to be inhibited (Additional file 1: Table S3). The main canonical pathways affected were atherosclerosis signaling (*p* = 3.2E−07), T helper cell differentiation (*p* = 6.6E−04), IL-6 signaling (*p* = 2.1E−03) (Additional file 1: Table S4).

Metformin treatment of CD34^+^ cells under hyperglycemia led to differential expression of 65 genes compared with hyperglycemia alone. Approximately 40 % of the genes were upregulated, and 60 % were downregulated. The top differentially expressed genes are listed in Additional file 1: Table S5. The top associated molecular functions predicted by IPA were cell death/survival (*p* = 1.29E−02), and cell proliferation/growth (*p* = 3.25E−03). The genes attributed to cell death/survival were killer cell immunoglobulin-like receptor, two domains, short cytoplasmic tail, 5 (*KIR2DS5*) 1.75-fold, *p* = 2.27E−02, ubiquitin specific peptidase 18 (*USP18*) 1.63-fold, *p* = 3.10E−03. The genes related to cell proliferation/growth were epithelial cell adhesion molecule (*EPCAM*) −2.06-fold, *p* = 9.93E−03, *USP18*, and hydroxy-delta-5-steroid dehydrogenase, 3 beta- and steroid delta-isomerase 1 (*HSD3B1*) −1.64-fold, *p* = 2.73E−02.

It is of particular interest that vascular endothelial growth factor receptor-2 (*VEGFR*-*2*) was downregulated under hyperglycemia in a dose-dependent manner (−1.95-fold, *p* = 3.23E−04) for 16.5 mmol/L and (−3.0-fold, *p* = 5.3E−06) for 25 mmol/L and not improved by metformin treatment (Additional file 1: Table S3).

### Metformin drives a gene expression profile consistent with pro-angiogenic action in CD34^+^ cells under hyperglycemia with hypoxia

We identified 1006 differentially expressed genes in cells under hyperglycemia exposed to hypoxia compared with hyperglycemia. 52 % of the genes were upregulated whereas 48 % were downregulated. The top 20 significantly differentially expressed genes are listed in Table 2. The most affected functions predicted by IPA were cell cycle (*p* = 1.95E−19), cellular movement (*p* = 2.03E−09), and cellular proliferation (*p* = 1.39E−07), which were predicted to be activated, whereas chemotaxis of vascular endothelial cells (*p* = 6.44E−03) was predicted to be inhibited. Downregulation of pro-angiogenic cytokines *IL*-*8* (−2.12-fold, *p* = 3.55E−04) and *HGF* (−1.64-fold, *p* = 2.33E−03) was detected, while upregulation of angiogenic inhibitors *TIMP1* (2.36-fold, *p* = 6.34E−06), *TIMP3* (2.15-fold, *p* = 2.29E−02), and *CXCL10* (1.89-fold, p = 2.04E−02) was indicated.

**Table 2:** Top differentially expressed genes in CD34^+^cells induced by hyperglycemia–hypoxia for 3 h versus hyperglycemia

Gene name	Gene symbol	*p* value	FC
Non-SMC condensin I complex, subunit H	NCAPH	1.46E−09	2.24
Polo-like kinase 1	PLK1	1.54E−09	4.71
Cyclin A2	CCNA2	1.94E−09	2.57
Serine/arginine-rich splicing factor 4	SRSF4	1.15E−08	−1.73
Protein regulator of cytokinesis 1	PRC1	1.17E−08	2.17
TIMP metallopeptidase inhibitor 3	TIMP3	1.24E−08	2.14
Lysine (K)-specific demethylase 5D	KDM5D	2.59E−08	1.86
TPX2, microtubule-associated, homolog (*Xenopus laevis*)	TPX2	6.63E−08	2.64
Cell division cycle 20 homolog (*S. cerevisiae*)	CDC20	8.42E−08	3.08
Olfactory receptor, family 2, subfamily L, member 13	OR2L13	9.20E−08	−1.93
Prohibitin	PHB	9.25E−08	1.99
ZW10 interactor	ZWINT	9.82E−08	1.93
X-ray repair complementing defective repair in Chinese hamster cells 2	XRCC2	1.44E−07	1.61
Brain expressed, X-linked 4	BEX4	1.47E−07	−1.76
Protein kinase, membrane associated tyrosine/threonine 1	PKMYT1	1.61E−07	2.14
Golgin A8 family, member B	GOLGA8B	1.62E−07	−1.64
Holliday junction recognition protein	HJURP	1.67E−07	2.55
S100 calcium binding protein A8	S100A8	2.10E−07	−5.24
TIMP metallopeptidase inhibitor 1	TIMP1	6.34E−06	2.36
Chemokine (C–X–C motif) ligand 10	CXCL10	2.04E−02	1.89

The gene list was created by importing Affymetrix .CEL files to Partek Genomic Suite version 6.6. The data were RMA normalized. Differentially expressed gene list was generated using one-way ANOVA, Benjamini and FDR-unadjusted *p* value <0.05 with a fold change cutoff of 1.5 was applied

Under metformin combined with hyperglycemia–hypoxia, 317 genes were differentially expressed versus no metformin. Forty-two percent of the genes were upregulated whereas 58 % were downregulated. The 20 most differentially expressed genes are listed in Table 3. The most affected functions predicted by IPA were cellular movement (*p* = 3.19E−05), and DNA replication and repair (*p* = 1.41E−04). The main canonical pathways affected were mitochondrial dysfunction (*p* = 2.0E−03), triacylglycerol biosynthesis (*p* = 7.1E−03), MAPK signaling (*p* = 7.9E−03), Type 1 DM signaling (*p* = 9.5E−03), and IL-8 signaling (*p* = 2.3E−02) (Additional file 1: Table S6).

**Table 3:** Top differentially expressed genes in CD34^+^ cells treated with hyperglycemia–metformin–hypoxia for 3 h versus hyperglycemia–hypoxia

Gene name	Gene symbol	*p* value	FC
Serine/arginine-rich splicing factor 4	SRSF4	3.32E−09	1.87
Brain expressed, X-linked 4	BEX4	1.43E−07	1.76
Dihydrouridine synthase 4-like (*S. cerevisiae*)	DUS4L	2.41E−07	1.60
Mitochondrial ribosomal protein S21	MRPS21	2.99E−07	1.52
Endoplasmic reticulum protein 29	ERP29	6.47E−07	−1.74
Prohibitin	PHB	1.40E−06	−1.67
Chromosome 11 open reading frame 58	C11orf58	1.48E−06	1.91
NOP14 nucleolar protein homolog (yeast)	NOP14	1.50E−06	1.52
Deoxynucleotidyltransferase, terminal, interacting protein 1	DNTTIP1	1.97E−06	1.91
Family with sequence similarity 98, member A	FAM98A	2.03E−06	1.52
Dynactin 3 (p22)	DCTN3	2.25E−06	−2.14
Solute carrier family 25, member 32	SLC25A32	2.47E−06	2.02
Heterogeneous nuclear ribonucleoprotein H3 (2H9)	HNRNPH3	4.21E−06	1.72
Glycerol-3-phosphate acyltransferase, mitochondrial	GPAM	4.31E−06	−10.76
Zinc finger protein 391	ZNF391	4.55E−06	1.59
Chromosome 11 open reading frame 51	C11orf51	4.61E−06	2.36
USO1 vesicle docking protein homolog (yeast)	USO1	4.64E−06	1.55
Transducer of ERBB2, 2	TOB2	1.22E−05	4.48
TIMP metallopeptidase inhibitor 1	TIMP1	3.90E−04	−1.68
Chemokine (C–X–C motif) ligand 10	CXCL10	1.28E−02	−2.01

Refer to legend in Table 2

Furthermore, metformin downregulated the angiogenic inhibitors *TIMP1* [30] (−1.68-fold, *p* = 3.90E−04), and *CXCL10* [31] (−2.01-fold, *p* = 1.28E−02), whilst had no effect on expression of pro-angiogenic factors under hyperglycemia–hypoxia versus no metformin.

### Confirmation of effects of metformin on gene expression in CD34^+^ cells using qRT-PCR

Seven pro-angiogenic factors *CCL2*, *CCL5*, *HGF*, *IL*-*1a*, *IL*-*6*, *IL*-*8*, selectin P (*SELP)* and two angiogenic inhibitors *CXCL10* and *TIMP1* with critical biological functions were validated by qRT-PCR (Additional file 1: Figure S3) and their results were compared to those obtained from the microarray experiments using the same RNA samples (Additional file 1: Figure S4). The qRT-PCR results were concordant with all gene expression changes indicated by the microarray data except *TIMP1* which was justified later (Additional file 1: Table S7). Moreover, hyperglycemia and metformin resulted in an increase of the mRNA levels of *HGF* (1.7-fold, *p* = 0.001), and *IL*-*6* (2.8-fold, *p* = 0.01) although this increase remained below normal level observed at euglycemia.

## Discussion

To our knowledge, this is the first report describing the effect of a physiological concentration of metformin on the angiogenic potential of CD34^+^ cells. Metformin is a hypoglycemic agent found to have cardioprotective properties as borne by a large clinical trial [14]. It has been shown that metformin has a protective effect against free fatty acid induced apoptosis [32] and its administration in diabetic patients prior to stroke onset was associated with reduced neurological severity and improved acute-phase therapy outcomes [33]. Moreover, metformin’s pleiotropic effects and cardioprotective role beyond glucose-lowering effect has been recently highlighted, encouraging implementation in prospective studies [34]. A recent study has shown that metformin monotherapy lead to an improvement in multiple clinical parameters and a reduction in all-cause mortality and CVD events in Type 2 DM patients [35] however, the underlying mechanism is unclear.

In diabetic patients CD34^+^ cells displayed a vasoreparative dysfunction due to impaired paracrine function and reduced sensitivity to hypoxia [10]. Whilst, the outcome of trials involving administration of angiogenic cytokines for the purpose of therapeutic angiogenesis as an alternative treatment for ischemic heart disease have been disappointing [36–38].

Autologous CD34^+^ based stem cell therapies have been pioneered as an alternative treatment for CVD [39–42]. Therefore, we studied vasoreparative properties of CD34^+^ cells following incubation with physiological concentration of metformin, under conditions of hyperglycemia, and/or hypoxia as a model of intervention window during acute myocardial infarction. Our research focused on the paracrine secretion of selected pro-inflammatory, pro-angiogenic factors and angiogenic inhibitors, which effect was functionally studied using an in vitro tube formation assay. Furthermore, gene expression profiling of CD34^+^ cells, treated under the above mentioned conditions, was performed focusing on pro-angiogenic mechanisms.

### Pro-angiogenic effect of metformin

We found that a physiological dose of metformin increased tube formation under the condition of hyperglycemia–hypoxia combined. This was associated with downregulation of the mRNA of the angiogenic inhibitors *CXCL10* and *TIMP1* observed by microarray analysis and further confirmed by qRT-PCR. The discrepancy in *TIMP1* results obtained from microarray data and qRT-PCR could be due to non-concordance of transcripts used in the microarray probe set and qRT-PCR [43]. Conversely, in the absence of metformin, *CXCL10* and *TIMP1* gene expression was significantly upregulated under hyperglycemia–hypoxia versus hyperglycemia. Our findings are concordant with clinical/laboratory data that demonstrated a suppression in angiogenesis due to an increase in angiogenic inhibitors in patients with diabetes combined with an increase in CXCL10 and TIMP1 expression at the transcriptional and protein levels [44, 45].

Under conditions of euglycemia and euglycemia-hypoxia, metformin had no measured effect on angiogenesis as both tube formation and gene expression of *CXCL10* and *TIMP1* were unchanged. Therefore, it appears that metformin has no additional pro-angiogenic effect in the non-diabetic state. This finding is in keeping with recent clinical studies, which revealed that metformin exhibited no effect on several surrogate markers of CVD in non-diabetic patients nor led to beneficial clinical outcome [46, 47].

In support of enhanced angiogenesis by metformin, we found that although the concentration of VEGFA was progressively increased under hyperglycemia–hypoxia it was further enhanced following metformin.

We confirmed in our in vitro model, as it was shown in clinical studies, that hyperglycemia alone increased VEGFA but downregulated VEGFR-2 gene expression, neither of which were affected by metformin. Our findings are concordant with another study in which VEGFA expression was found to be increased in myocardial tissue of diabetic patients, whilst a decrease in VEGF receptors was documented leading to downregulation of VEGF signal transduction [48].

**Fig. 3:**
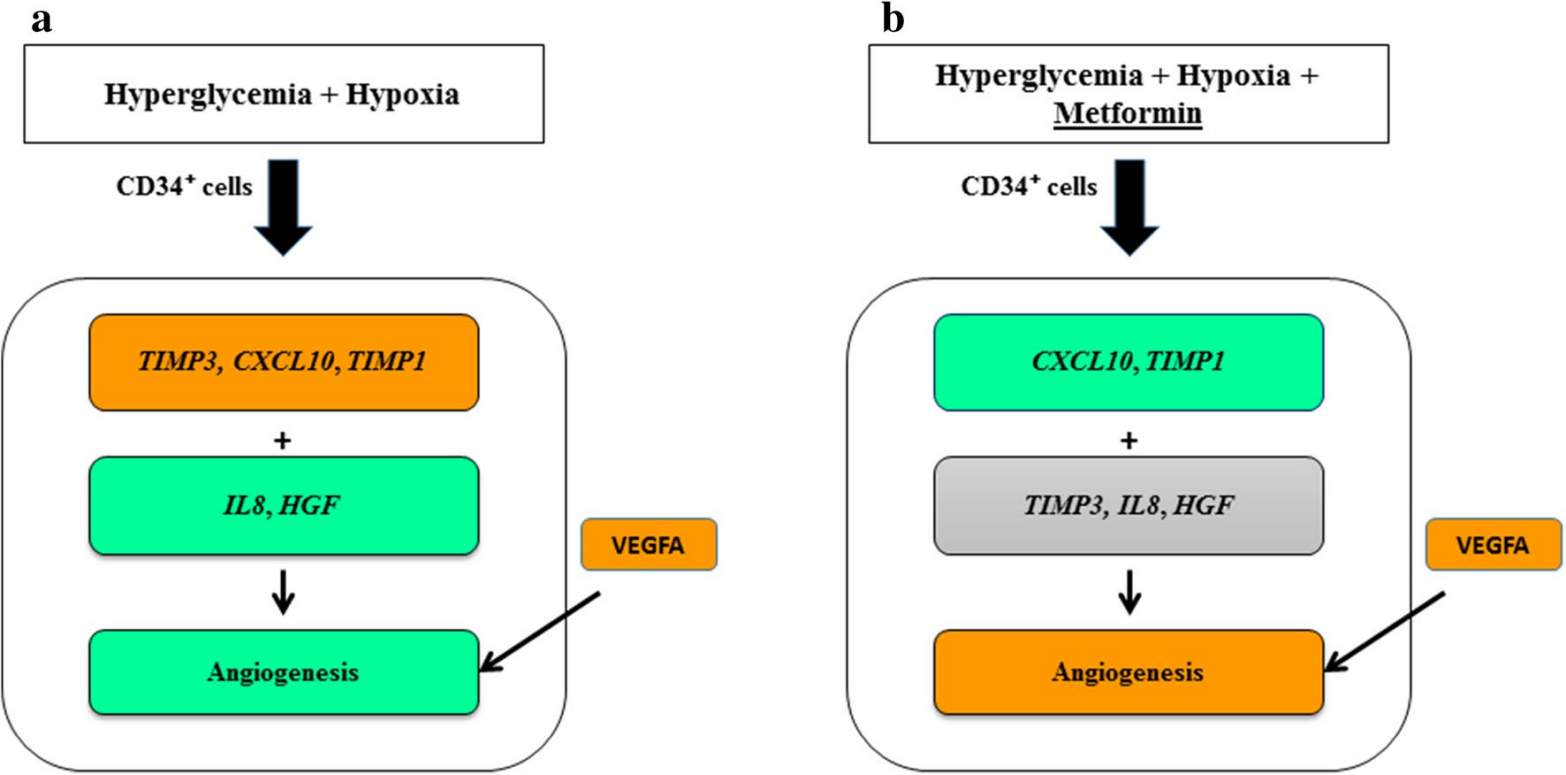
Summary of the effect of metformin on CD34^+^ cells incubated with hyperglycemia–hypoxia. CD34^+^ cells were treated with hyperglycemia–hypoxia versus euglycemia (**a**), and hyperglycemia–hypoxia with metformin versus metformin untreated-condition (**b**). *Green shading* indicates downregulation, *orange shading* indicate upregulation and *gray shading* indicate unchanged gene expression. In vitro tube formation (angiogenesis), *green shading* indicates inhibition, whereas *orange shading* indicates activation

Thus, although metformin further increased angiogenic factor, VEGFA, we have shown that the cardioprotection offered was predominantly by reducing angiogenic inhibitors; *CXCL10* and *TIMP*-*1*; rather than further increasing VEGFA alone. This means that the process of angiogenesis is under a fine balance of pro and anti-angiogenic factors to ensure tightly regulated process. Consequently, in the condition of diabetes combined with hypoxia it is not sufficient to further increase proangiogenic factors alone, but downregulation of inhibitors is required for altering the balance towards angiogenesis (Fig. 3).

### Pro-inflammatory effect of metformin

It has been assumed that mechanism behind CD34^+^ cells based therapy for CVD is mediated by paracrine function. Thus, we hypothesized that metformin improved the paracrine function of CD34^+^ cells. However, we found very low protein levels of IL-1β, IL-6, and TNF-α with non-significantly reduced levels of IL-8 expression in CD34^+^ cell CM in all studied conditions including metformin. These findings can be explained by downregulation of genes coding for inflammatory cytokines in CD34^+^ cells under metformin and euglycemia, euglycemia-hypoxia, and hyperglycemia.

We have shown that under euglycemia, metformin displayed an inhibitory effect on genes of pro-inflammatory factors *CCL2*, *CCL5*, *CD226*, *IL*-*1α*, *IL*-*6*, *IL*-*8*, and Integrin, beta 3 (*ITGB3*) whilst upregulating *STEAP* family member 4 (*STEAP4*). We thus believe that the anti-inflammatory action of metformin could be mediated by augmenting mRNA expression of *STEAP4*. This anti-inflammatory effect has been previously demonstrated in fibroblast-like synoviocytes by upregulation of STEAP4 leading to the suppression of IL-6 and IL-8 expression [49]. In concordance with our results another study in smooth muscle cells, ECs, or macrophages has shown that a physiological dose of metformin inhibited the expression of IL-1β, IL-6, and IL-8 [50].

Furthermore, the same mechanism of inhibition of pro-inflammatory cytokines was demonstrated under euglycemia-hypoxia with the increase in *STEAP4* expression, while metformin had no additional effect on those genes.

These data suggest that metformin and/or hypoxia in CD34^+^ cells are beneficial in suppressing the inflammatory response. In fact, low oxygen tension in the range of 3–5 % (hypoxia) is the preferable environment for stem cells (including CD34^+^ cells) to reside quiescent in their niches and maintain pluripotency with no effect on proliferation [51].

In addition, we have established that *TOB2* which acts as an anti-proliferative factor [52] was upregulated under hypoxic condition but was inhibited by metformin. TOB2 is a member of the mammalian BTG/TOB family of anti-proliferative proteins [53]. TOB2 were previously detected to be expressed in mouse embryonic stem cells that play a critical role in the maintenance of stem cells properties [52].

Similarly, under hyperglycemia and hyperglycemia–hypoxia our transcriptome analysis demonstrated downregulation of pro-inflammatory and pro-angiogenic cytokines, chemokines and their receptors with no additional effect of metformin. The failure to detect the suppressive effect of metformin on those cells is possibly due to already maximally suppressed cytokine gene expression.

We studied pro-inflammatory paracrine secretion by CD34^+^ cells without cell co-culture or cell expansion. This may be a limitation of our approach as we underestimate the interactions of CD34^+^ cells with other cells known to induce cytokine secretion in co-culture [54] or cytokine secretion from expanded CD34^+^ cells [55]. Specifically, the co-culture of CD34^+^ cells with HUVEC induced paracrine secretion of pro-inflammatory cytokines including IL-8, IL-6, CCL2, CCL3, and CCL4 and angiogenesis under hypoxia [54]. Also, the co-culture of CD34^+^ and CD34^−^ cells lead to enhancement of tube formation and cell migration compared with the culture of CD34^+^ cells alone through the cell to cell interactions and paracrine effects [56]. Furthermore, it has been reported that macrophages migrate to hypoxic areas that stimulate expression of pro-angiogenic such as VEGFA and pro-inflammatory genes in an attempt to repair the damage [57]. Together these data demonstrate the significant role played by CD34^−^ cells in interaction with CD34^+^ cells to improve tissue repair.

It is envisaged that the findings from our study will be validated in in vivo settings using suitable animal models with CXCL10 and/or TIMP1 overexpression. However, the current lack of appropriate animal models make it impossible at present to confirm the suggested mechanism in vivo. Moreover, it will be of importance if these findings can also be proven in CD34^+^ cells obtained from diabetic patients.

## Conclusions

In conclusion, vascular protection by metformin is mediated by its dual effect to enhance the angiogenic potential of CD34^+^ cells by suppressing several angiogenic inhibitors including *CXCL10* and *TIMP1* whilst further increasing VEGFA secretion. Those angiogenic inhibitors are potential therapeutic targets for CVD interventions in diabetes.

## Additional file

10.1186/s12933-016-0344-2 Cell viability assay on HUVEC treated with sunitinib. **Figure S2.** Effect of metformin on euglycemia-hypoxia treated CD34^+^ cells. **Figure S3.** Validation of selected pro-angiogenic factors and angiogenic inhibitors in CD34^+^ cells by qRT-PCR. **Figure S4.** Heatmap of the pro-angiogenic and angiogenic inhibitors in CD34^+^ stem cells. **Table S1.** Effect of 3 h hypoxia on biological functions involved in CD34^+^ cells. **Table S2.** Top 30 differentially expressed genes in CD34^+^ cells induced by hyperglycemia compared to control. **Table S3.** Top biological functions involved in CD34^+^ cells cultured in hyperglycemia. **Table S4.** Top canonical pathways involved in CD34^+^ cells cultured in hyperglycemia. **Table S5.** Top 30 differentially expressed genes in CD34^+ ^cells induced by hyperglycemia and treated with metformin compared to hyperglycemia. **Table S6.** Effect of metformin on canonical pathways involved in CD34^+^ cells cultured in combined hyperglycemia-hypoxia for 3 h. **Table S7.** Comparison of microarray and real-time PCR data on CD34^+^ cells treated with hypoxia, hyperglycemia and hyperglycemia-hypoxia in the presence and absence of metformin.

## Abbreviations

C3: complement 3
CCL(number): chemokine (C–C motif) ligand (number)
CM: conditioned medium
CVD: cardiovascular disease
CXCL10: chemokine (C–X–C motif) ligand 10
DM: diabetes mellitus
EPCAM: epithelial cell adhesion molecule
HGF: hepatocyte growth factor
HSD3B1: hydroxy-delta-5-steroid dehydrogenase, 3 bet- and steroid delta-isomerase 1
HUVEC: human umbilical vein endothelial cell
IL-(number): interleukin-(number)
ITGB3: integrin, beta 3
KIR2DS5: killer cell immunoglobulin-like receptor, two domains, short cytoplasmic tail, 5
MI: myocardial infarction
MT2A: metallothionein 2A
SELP: selectin P
SPINK7: serine peptidase inhibitor, Kazal type 7
STEAP4: STEAP family member 4
TIMP1: tissue inhibitor of metalloproteinase 1
TOB2: transducer of ERBB2, 2
UCB: umbilical cord blood
USP18: ubiquitin specific peptidase 18
VEGFA: vascular endothelial growth factor A
