# Supplementary material for: Metformin improves the angiogenic potential of human CD34+ cells co-incident with downregulating CXCL10 and TIMP1 gene expression and increasing VEGFA under hyperglycemia and hypoxia within a therapeutic window for myocardial infarction
Source: Cardiovasc Diabetol. 2016 Feb 9;15:27. doi: 10.1186/s12933-016-0344-2 (PMC4748498; doi:10.1186/s12933-016-0344-2)
Supplement: Supplementary file 1 — 10.1186/s12933-016-0344-2 Cell viability assay on HUVEC treated with sunitinib. Figure S2. Effect of metformin on euglycemia-hypoxia treated CD34+ cells. Figure S3. Validation of selected pro-angiogenic factors and angiogenic inhibitors in CD34+ cells by qRT-PCR. Figure S4. Heatmap of the pro-angiogenic and angiogenic inhibitors in CD34+ stem cells. Table S1. Effect of 3 h hypoxia on biological functions involved in CD34+ cells. Table S2. Top 30 differentially expressed genes in CD34+ cells induced by hyperglycemia compared to control. Table S3. Top biological functions involved in CD34+ cells cultured in hyperglycemia. Table S4. Top canonical pathways involved in CD34+ cells cultured in hyperglycemia. Table S5. Top 30 differentially expressed genes in CD34+ cells induced by hyperglycemia and treated with metformin compared to hyperglycemia. Table S6. Effect of metformin on canonical pathways involved in CD34+ cells cultured in combined hyperglycemia-hypoxia for 3 h. Table S7. Comparison of microarray and real-time PCR data on CD34+ cells treated with hypoxia, hyperglycemia and hyperglycemia-hypoxia in the presence and absence of metformin. [file 12933_2016_344_MOESM1_ESM.pdf]

Online Supplemental Materials

Metformin Improves the Angiogenic Potential of Human CD34$^+$ Cells Co-incident with Downregulating *CXCL10* and *TIMP1* Gene Expression and Increasing VEGFA under Hyperglycemia and Hypoxia within a Therapeutic Window for Myocardial Infarction

Sherin Bakhashab[1,3,4], Fahad W. Ahmed[1,2], Hans-Juergen Schulten[4], Ayat Bashir[1], Sajjad Karim[4], Abdulrahman L. Al-Malki[3], Mamdooh A. Gari[4], Adel M. Abuzenadah[4], Adeel G. Chaudhary[4], Mohammed H. Alqahtani[4], Sahira Lary[3], Farid Ahmed[4], Jolanta U. Weaver[1,2*]

[1]*Institute of Cellular Medicine, Newcastle University, Newcastle Upon Tyne, UK*

[2]*Queen Elizabeth Hospital, Gateshead, Newcastle Upon Tyne, UK*

[3]*Biochemistry Department, King Abdulaziz University, Jeddah, Saudi Arabia*

[4]*Centre of Excellence in Genomic Medicine Research, King Abdulaziz University, Jeddah, Saudi Arabia*

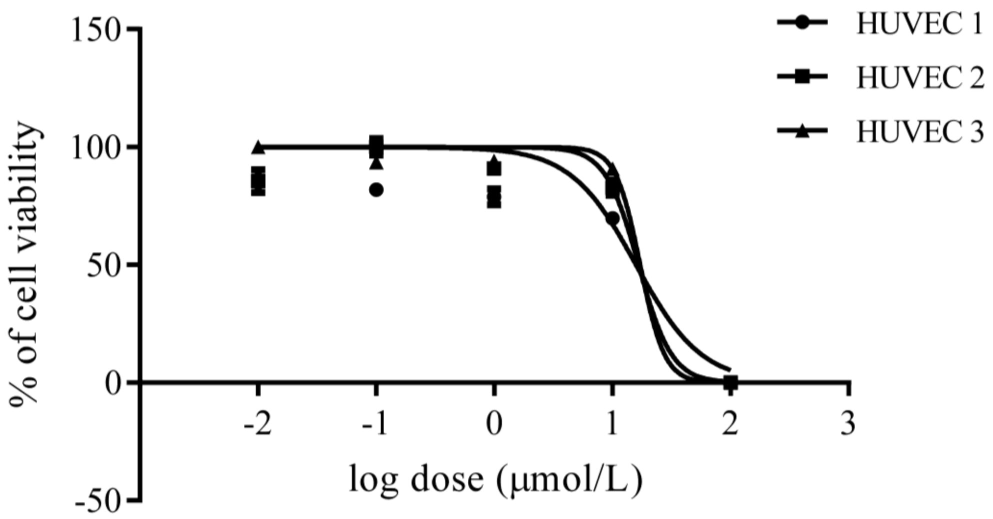

Supplementary Figure 1: Cell viability assay on HUVEC treated with sunitinib. HUVEC (10^4 cells/ well) in 96 well plate were treated with increasing concentrations of sunitinib (0.01, 0.1, 1.0, 10, and 100 µmol/L) in quadruplicates. After 6 hours CellTiter-Blue reagent (Promega) was added to each well, and the cells were incubated for 2 hours prior to recording fluorescence (560/590 nm). Results are presented as mean ± SEM. The mean of IC50 was calculated as 16.6 µmol/L.

Supplementary Table 1: Effect of 3 hours hypoxia on biological functions involved in $CD34^+$ cells.

Functions Annotation	*p*-Value	Predicted Activation State	Activation z-score	Molecules	Number of Molecules
Inflammatory Response	1.09E-11	Decreased	-2.725	↓AQP9, ↓C3, ↓CCL2, ↓CCL20, ↓CCL5, ↓CCR7, ↓CD14, ↓CXCL5, ↓FOS, ↓IL-1α, ↓IL-6, ↓IL-8, ↓mir-21, ↓PLA2G7, ↓PPBP, ↓RGS1, ↑S100A8, ↓TNFAIP6, ↓UTS2	19
Cellular Movement	2.82E-11	Decreased	-2.274	↓AQP9, ↓C3, ↓CCL2, ↓CCL20, ↓CCL5, ↓CCR7, ↓CXCL5, ↓IL-1α, ↓IL-8, ↓PLA2G7, ↓PPBP, ↓RGS1, ↑S100A8, ↓UTS2	14
Cell-To-Cell Signaling and Interaction	4.69E-11	Decreased	-3.289	↓C3, ↓CCL2, ↓CCL5, ↓CCR7, ↓CD14, ↓CLEC1B, ↓CTSL1, ↓CXCL5, ↓DUSP1, ↓FOS, ↓IL-1α, ↓IL-6, ↓IL-8, ↓KRT18, ↓MET, ↓ mir-21, ↓MS4A1, ↓NCOR2, ↑PPBP, ↑S100A8	20
Cellular Growth and Proliferation	3.92E-08	Decreased	-2.195	↓CCL2, ↓CCL5, ↓DUSP1, ↓FOS, ↓IL-1α, ↓IL-6, ↓IL-8, ↓MET, ↓mir-21, ↓TFPI2, ↑TOB2, ↓UTS2	12

Gene list was generated by using IPA software. The activation z-score was used in the calculation of significant changes in gene expression in different samples and conditions. It is calculated from the dataset and indicates activation or inhibition of the biological function as (+) indicates activation while (-) indicates inhibition. The arrow ↑ indicates gene is upregulated and ↓ indicates gene is downregulated. Key: *AQP9*: aquaporin 9; *C3*: complement component 3; *CCL2*: chemokine (C-C motif) ligand 2; *CCR7*: chemokine (C-C motif) receptor 7; *CD14*: CD14 molecule; *CLEC1B*: C-type lectin domain family 1, member B; *CTSL1*: cathepsin L1; *CXCL5*: chemokine (C-X-C motif) ligand 5; *DUSP1*: dual specificity phosphatase 1; *FOS*: FBJ murine osteosarcoma viral oncogene homolog; *IL-1α*: interleukin 1 alpha; *KRT18*: keratin 18; *MET*: met proto-oncogene (hepatocyte growth factor receptor); *mir-21*: microRNA 21; *MS4A1*: membrane-spanning 4-domains, subfamily A, member 1; *NCOR2*: nuclear receptor corepressor 2; *PLA2G7*: phospholipase A2, group VII (platelet-activating factor acetylhydrolase, plasma); *PPBP*: pro-platelet basic protein (chemokine (C-X-C motif) ligand 7); *RGS1*: regulator of G-protein signaling 1; *S100A8*: S100 calcium binding protein A8;*TFPI2*: tissue factor pathway inhibitor 2; *TNFAIP6*: tumour necrosis factor, alpha-induced protein 6; *TOB2*: transducer of ERBB2, 2; *UTS2*: urotensin 2.

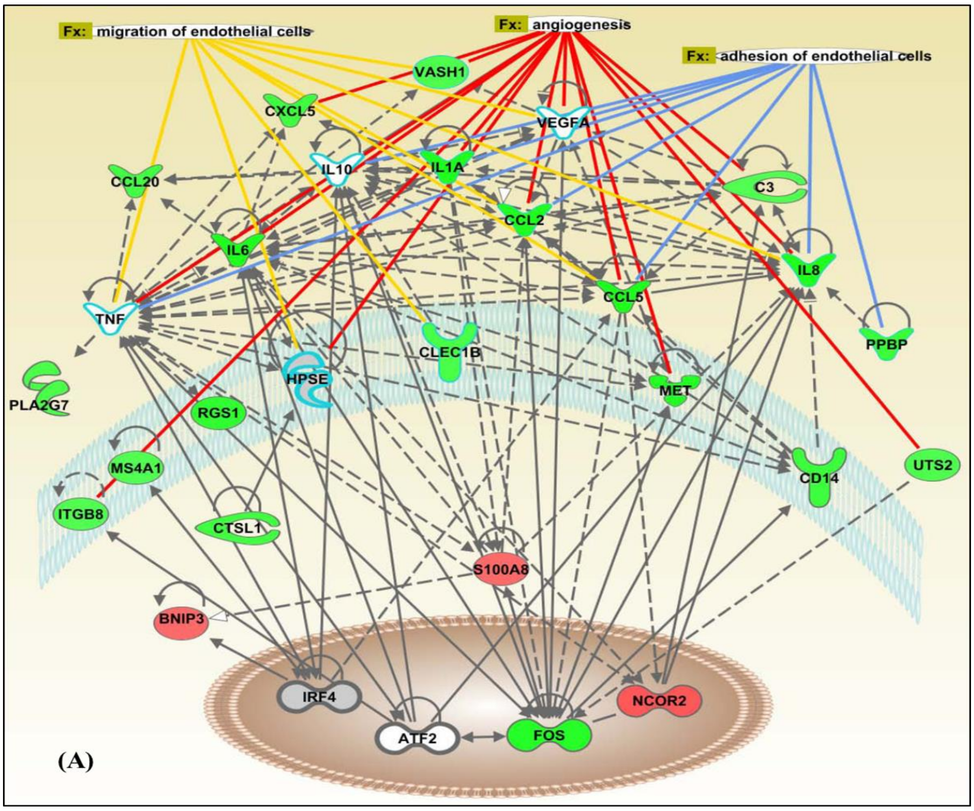

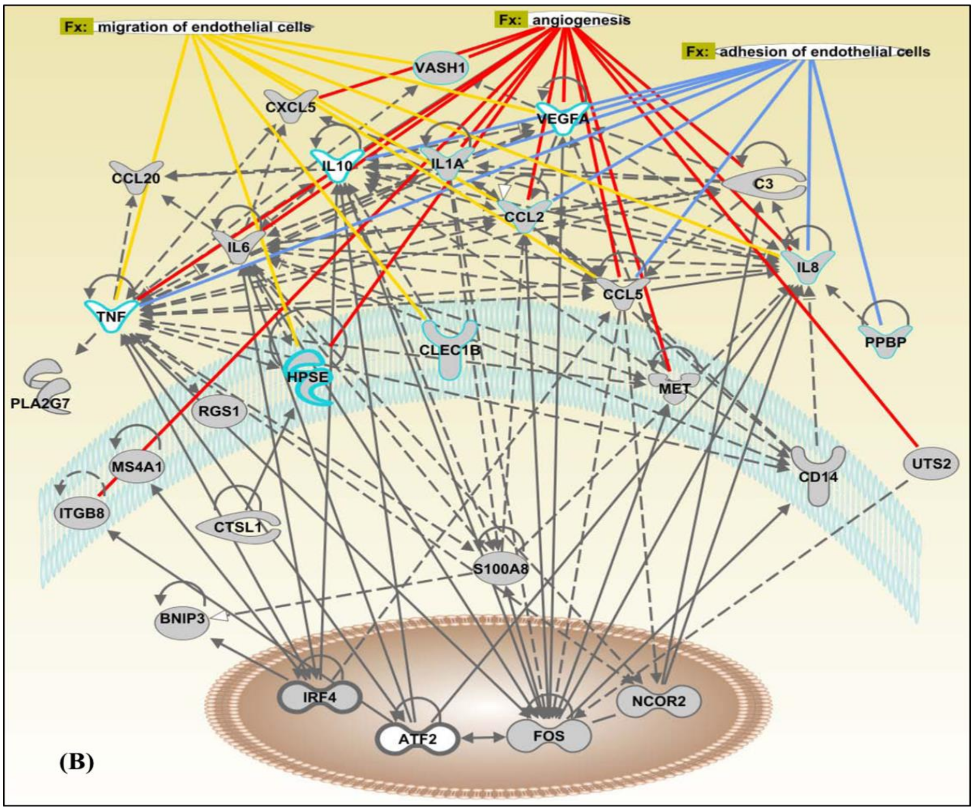

Gene Name	Gene symbol	FC	Family
Activating transcription factor 2	ATF2		transcription regulator
BCL2/adenovirus E1B 19kDa interacting protein 3	BNIP3	1.51	other
Complement component 3	C3	-1.51	peptidase

Chemokine (C-C motif) ligand 2	CCL2	-2.25	cytokine
Chemokine (C-C motif) ligand 20	CCL20	-1.64	cytokine
Chemokine (C-C motif) ligand 5	CCL5	-2.29	cytokine
CD14 molecule	CD14	-1.71	transmembrane receptor
C-type lectin domain family 1, member B	CLEC1B	-1.60	transmembrane receptor
Cathepsin L1	CTSL1	-1.67	peptidase
Chemokine (C-X-C motif) ligand 5	CXCL5	-1.68	cytokine
FBJ murine osteosarcoma viral oncogene homolog	FOS	-1.89	transcription regulator
Heparanase	HPSE	-1.44	enzyme
Interleukin 10			cytokine
Interleukin 1, alpha	IL1A	-4.67	cytokine
Interleukin 6 (interferon, beta 2)	IL6	-2.50	cytokine
Interleukin 8	IL8	-2.43	cytokine
Interferon regulatory factor 4	IRF4	-1.14	transcription regulator
Integrin, beta 8	ITGB8	-1.52	other
Met proto-oncogene (hepatocyte growth factor receptor)	MET	-1.91	kinase
Membrane-spanning 4-domains, subfamily A, member 1	MS4A1	-1.50	other
Nuclear receptor corepressor 2	NCOR2	1.70	transcription regulator
Phospholipase A2, group VII (platelet-activating factor acetylhydrolase, plasma)	PLA2G7	-1.59	enzyme
Pro-platelet basic protein (chemokine (C-X-C motif) ligand 7)	PPBP	-1.74	cytokine
Regulator of G-protein signaling 1	RGS1	-1.81	other
S100 calcium binding protein A8	S100A8	1.52	other
Tumour necrosis factor			cytokine
Urotensin 2	UTS2	-1.53	other
Vasohibin 1	VASH1	-1.55	other
Vascular endothelial growth factor A	VEGFA		growth factor

Supplementary Figure 2: Effect of metformin on euglycaemia-hypoxia treated CD34$^+$ cells. CD34$^+$ cells were treated with euglycaemia and hypoxia for 3 hours in the presence or absence of metformin for 24 hours. A set of differentially expressed genes was generated by comparing (A) euglycaemia and hypoxia versus euglycaemia or (B) euglycaemia and metformin exposed to hypoxia versus euglycaemia and hypoxia without metformin. The comprehensive network was created by IPA software. Green shades indicate downregulated, red shades indicate upregulated genes and grey unchanged genes.

Supplementary Table 2: Top 30 differentially expressed genes in CD34$^+$cells induced by hyperglycaemia compared to control.

Gene Name	Gene Symbol	*p*-value	FC
Lysine (K)-specific demethylase 5D	KDM5D	2.32E-11	-3.56
Selectin P (granule membrane protein 140kDa, antigen CD62)	SELP	3.27E-09	-5.01
Gamma-aminobutyric acid (GABA) A receptor, epsilon	GABRE	3.31E-09	-2.76
Integrin, beta 3 (platelet glycoprotein IIIa, antigen CD61)	ITGB3	3.71E-09	-6.70
Chromosome Y open reading frame 15B	CYorf15B	3.74E-09	-5.06
Coagulation factor XIII, A1 polypeptide	F13A1	4.77E-09	-3.50
Polycystic kidney and hepatic disease 1 (autosomal recessive)-l	PKHD1L1	6.50E-09	-6.43
KIAA0087	KIAA0087	1.40E-08	2.48
Chromosome Y open reading frame 15A	CYorf15A	2.26E-08	-4.54
DEAD (Asp-Glu-Ala-Asp) box polypeptide 3, Y-linked	DDX3Y	2.29E-08	-4.16
Ubiquitously transcribed tetratricopeptide repeat gene, Y-linked	UTY	2.94E-08	-3.89
Heparanase	HPSE	3.39E-08	-2.81
Nexilin (F actin binding protein)	NEXN	9.83E-08	-3.21
Major histocompatibility complex, class II, DQ beta 1	HLA-DQB1	1.03E-07	-7.01
Ubiquitin specific peptidase 9, Y-linked	USP9Y	1.19E-07	-3.83
Major histocompatibility complex, class II, DQ beta 1	HLA-DQB1	1.44E-07	-8.74
Galectin-related protein	HSPC159	1.55E-07	-5.74
Epithelial cell adhesion molecule	EPCAM	2.04E-07	-2.96
Zinc finger protein, Y-linked	ZFY	2.10E-07	-3.23
Dynamin 3	DNM3	2.35E-07	-2.06
Eukaryotic translation initiation factor 1A, Y-linked	EIF1AY	3.09E-07	-4.85
Phosphodiesterase 3A, cGMP-inhibited	PDE3A	3.98E-07	-2.52
Olfactory receptor, family 3, subfamily A, member 2	OR3A2	4.25E-07	2.31
Snail homolog 2 (Drosophila)	SNAI2	4.51E-07	2.16
Glycoprotein Ib (platelet), alpha polypeptide	GP1BA	6.25E-07	-2.70
Testis-specific transcript, Y-linked 10 (non-protein coding)	TTTY10	6.58E-07	-2.58
Latent transforming growth factor beta binding protein 1	LTBP1	6.69E-07	-2.94
C-type lectin domain family 1, member B	CLEC1B	1.06E-06	-4.25
Potassium channel, subfamily K, member 17	KCNK17	1.17E-06	2.55
Immunoglobulin lambda joining 3	IGLJ3	1.76E-06	2.31

The gene list was created by importing Affymetrix .CEL files to Partek Genomic Suite version 6.6. The data were RMA normalized. Differentially expressed gene list was generated using one-way ANOVA, FDR-unadjusted p-value < 0.05 with a fold change cutoff of 1.5 was applied.

Supplementary Table 3: Top biological functions involved in CD34⁺cells cultured in hyperglycaemia.

Functions	*p*-Value	Predicted Activation State	Activation z-score	Molecules	Number of Molecules
Cell-To-Cell Signaling and Interaction	1.80E-17	Decreased	-2.27	↓AVPR1A, ↓BDNF, ↑BLNK, ↑BTLA, ↓CCL2, ↑CCR7, ↓CD14, ↓CD226, ↓CD24, ↓CD36, ↑CD3E, ↑CD79A, ↓CLEC1B, ↓CTSL1, ↓CXCL2, ↓FCER1A, ↓GP1BA, ↓GP5, ↓GP6, ↑HCK, ↓HLA-DQA1, ↓HLA-DQB1, ↓HPSE, ↑IGHM, ↑IGK, ↓IL-18, ↓IL-1α, ↓IL-6, ↓IL-8, ↑IRF4, ↓ITGA2B, ↓ITGB3, ↓VEGFR-2, ↓LTBP1, ↓MET, ↑MS4A1, ↑NCOR2, ↑NCR1, ↓NTS, ↓PF4, ↑PLD2, ↓PPBP, ↓PRKCA, ↓PROS1, ↓PTGS2, ↓PTPRJ, ↑S100A12, ↑S100A8, ↓SELP, ↑SH2D1A, ↑TCL1A, ↓THBS1, ↓TLR4, ↑TLR7, ↑TNFRSF17, ↓VIP, ↓VWF	57
Cellular Growth and Proliferation	1.21E-12	Decreased	-1.40	↑BLNK, ↑BTLA, ↓CCL2, ↑CCR7, ↓CD14, ↓CD226, ↓CD24, ↓CD36, ↑CD3E, ↑CD79A, ↓CLECL1B, ↓CXCL2, ↓FYB, ↑HCK, ↓HLA-DQA1, ↓HLA-DQB1, ↑IGHM, ↑IGKC, ↑IKZF3, ↓IL-18, ↓IL-1α, ↓IL-5RA, ↓IL-6,IL-8, ↓INHBA, ↑IRF4, ↓ITGA2B, ↓ITGB3, ↓LTBP1, ↑LY9, ↑MS4A1, ↓PDE5A, ↓PF4, ↓PTGS2, ↓PTPRJ, ↓RUNX1T1, ↑SH2D1A, ↑SNAI2, ↑TCL1A, ↓THBS1, ↓TLR4, ↑TLR7, ↑TNFRSF17, ↓VIP	44
Inflammatory Response	1.59E-12	Decreased	-1.18	↑BLNK, ↑BTLA, ↓CCL2, ↑CCR7, ↓CD14, ↓CD226, ↑CD3E, ↑CD79A, ↓CLEC1B, ↓CTSL1, ↑HCK, ↓HLA-DQA1, ↓HLA-DQB1, ↓HPSE, ↑IGHM, ↓IL-18, ↓IL-1α, ↓IL-6, ↓IL-8, ↑IRF4, ↓LTBP1, ↓MET, ↑NCOR2, ↑NCR1, ↓PF4, ↑PLD2, ↓PRKCA, ↓PROS1, ↓PTGS2, ↓PTPRJ, ↑S100A12, ↑S100A8, ↑SH2D1A, ↑TCL1A, ↓THBS1, ↓TLR4, ↑TLR7, ↑TNFRSF17, ↓VIP	39
Cellular Movement	2.60E-11	Decreased	-3.31	↓AQP9, ↓BDNF, ↑BTLA, ↓CCL2, ↑CCR7, ↓CD14, ↓CD226, ↑CD36, ↓CD3E, ↓CLEC1B, ↓CTTN, ↓CXCL2, F13A1, ↓FCER1A, ↓FYB, ↓GP1BA, ↓GP6, ↑HCK, ↓IL-18, ↓IL-1α, ↓IL-6, ↓IL-8, ↓INHBA, ↓ITGA2B, ↓ITGB3, ↓VEGFR-2, ↓PF4, ↓PLA2G7, ↓PPBP, ↓PRKCA, ↓PTGS2, ↓PTPRJ, ↓S100A10, ↑S100A12, ↑S100A8, ↓SELP, ↑SH2D1A, ↓THBS1, ↓TLR4, ↑TLR7, ↑TNFSF8, ↓UTS2, ↓VIP, ↓VWF	44

The affected biological functions were generated by analysis of the gene list using IPA software. The activation z-score was used in the calculation of significant changes in gene expression in different samples and conditions. It is calculated from the dataset and indicates activation or inhibition of the biological function as (+) indicates activation while (-) indicates inhibition. The arrow ↑ indicates gene is upregulated and ↓ indicates gene is downregulated. Key: *AQP9*: aquaporin 9; *AVPR1A*: arginine vasopressin receptor 1A; *BDNF*: brain-derived neurotrophic factor; *BLNK*: B-cell linker; *BTLA*: B and T lymphocyte associated; *CCL2*: chemokine (C-C motif) ligand 2; *CCR7*: chemokine (C-C motif) receptor 7; *CD14*: CD14 molecule; *CD226*: CD226 molecule; *CD3E*: CD3e molecule, epsilon (CD3-TCR complex); *CD79A*: CD79a molecule, immunoglobulin-associated alpha; *CLEC1B*: C-type lectin domain family 1, member B; *CTSL1*: cathepsin L1; *CXCL2*: chemokine (C-X-C motif)

ligand 2; *F13A1*: coagulation factor XIII, A1 polypeptide; *FCER1A*: Fc fragment of IgE, high affinity I, receptor for; alpha polypeptide; *FYB*: FYN binding protein; *GP1BA*: glycoprotein Ib (platelet), alpha polypeptide; *GP5*: glycoprotein V (platelet); *HCK*: hemopoietic cell kinase; *HLA-DQA1*: major histocompatibility complex, class II, DQ alpha 1; *HPSE*: heparanase; *IGHM*: immunoglobulin heavy constant mu; *IGK*: immunoglobulin kappa locus; *IKZF3*: IKAROS family zinc finger 3 (Aiolos); *IL-18*: interleukin 18; *INHBA*: inhibin, beta A; *IRF4*: interferon regulatory factor 4; *ITGA2B*: integrin, alpha 2b (platelet glycoprotein IIb of IIb/IIIa complex, antigen CD41); *ITGB3*: integrin, beta 3 (platelet glycoprotein IIIa, antigen CD61); *LTBP1*: latent transforming growth factor beta binding protein 1; *LY9*: lymphocyte antigen 9; *MET*: met proto-oncogene (hepatocyte growth factor receptor); *MS4A1*: membrane-spanning 4-domains, subfamily A, member 1; *NCOR2*: nuclear receptor corepressor 2; *NCR1*: natural cytotoxicity triggering receptor 1; *NTS*: neurotensin; *PDE5A*: phosphodiesterase 5A, cGMP-specific; *PF4*: platelet factor 4; *PLA2G7*: phospholipase A2, group VII (platelet-activating factor acetylhydrolase, plasma); *PLD2*: phospholipase D2; *PPBP*: pro-platelet basic protein (chemokine (C-X-C motif) ligand 7); *PRKCA*: protein kinase C, alpha; *PROS1*: protein S (alpha); *PTGS2*: prostaglandin-endoperoxide synthase 2 (prostaglandin G/H synthase and cyclooxygenase); *PTPRJ*: protein tyrosine phosphatase, receptor type, J; *RUNX1T1*: runt-related transcription factor 1; translocated to, 1 (cyclin D-related); *S100A12*: S100 calcium binding protein A12; *SELP*: selectin P (granule membrane protein 140kDa, antigen CD62); *SH2D1A*: SH2 domain containing 1A; *SNAI2*: snail homolog 2 (Drosophila); *TCL1A*: T-cell leukemia/lymphoma 1A; *THBS1*: thrombospondin 1; *TLR4*: toll-like receptor 4; *TNFRSF17*: tumour necrosis factor receptor superfamily, member 17; *UTS2*: urotensin 2; *VEGFR-2*: vascular endothelial growth factor receptor-2; *VIP*: vasoactive intestinal peptide; *VWF*: von Willebrand factor.

Supplementary Table 4: Top canonical pathways involved in CD34$^+$ cells cultured in hyperglycaemia.

Ingenuity Canonical Pathways	*P*-value	Molecules
Atherosclerosis Signaling	3.2E-07	↓IL8, ↓IL1A, ↓IL18, ↓CCL2, ↓SELP, ↓CD36, ↓ALOX12, ↑S100A8, ↓IL6, ↓PLA2G7
T Helper Cell Differentiation	6.6E-04	↓IL18, ↓HLA-DQA1, ↑HLA-DOB, ↓HLA-DQB1, ↓IL6, ↓HLA-DRB5
IL-6 Signaling	2.1E-03	↓IL8, ↓IL1A, ↓IL18, ↓CD14, ↓IL6, ↓TNFAIP6

The arrow ↑ indicates gene is upregulated and ↓ indicates gene is downregulated. For genes key refer to Supplementary Table 3 legend.

Supplementary Table 5: Top 30 differentially expressed genes in $CD34^{+}$cells induced by hyperglycaemia and treated with metformin compared to hyperglycaemia.

Gene Name	Gene Symbol	*p*-value	FC
Small nucleolar RNA, H/ACA box 70C (retrotransposed)	SNORA70C	1.41E-03	1.95
HFM1, ATP-dependent DNA helicase homolog (S. cerevisiae)	HFM1	1.73E-03	-1.62
Eyes shut homolog (Drosophila)	EYS	2.10E-03	-1.53
Small Cajal body-specific RNA 16	SCARNA16	2.13E-03	-1.51
Ribosomal protein S6 pseudogene 6	RPS6P6	2.91E-03	1.79
Small nucleolar RNA, H/ACA box 71C	SNORA71C	2.99E-03	-1.59
Transmembrane protein 45A	TMEM45A	3.00E-03	-1.52
Ubiquitin specific peptidase 18	USP18	3.10E-03	1.63
Small nucleolar RNA, C/D box 94	SNORD94	3.94E-03	-1.51
Microfibrillar associated protein 5	MFAP5	5.58E-03	-1.54
Small nucleolar RNA, H/ACA box 68	SNORA68	5.69E-03	-1.60
CD177 molecule	CD177	5.72E-03	-1.86
Nuclear transport factor 2-like	LOC128322	6.53E-03	1.58
NADH dehydrogenase (ubiquinone) 1 alpha subcomplex, 4, 9kDa	NDUFA4	6.77E-03	-1.53
Family with sequence similarity 72, member D	FAM72D	7.17E-03	-1.64
SPANX family, member E	SPANXE	7.19E-03	1.58
Small Cajal body-specific RNA 6	SCARNA6	8.70E-03	-1.89
Heat shock protein 90kDa alpha (cytosolic), class A member 6	HSP90AA6P	9.46E-03	2.34
Chromosome 4 open reading frame 11	C4orf11	9.60E-03	1.58
Epithelial cell adhesion molecule	EPCAM	9.93E-03	-2.06
Golgin A6 family, member A	GOLGA6A	9.94E-03	1.89
Small nucleolar RNA, C/D box 15A	SNORD15A	1.04E-02	-1.52
Coiled-coil domain containing 122	CCDC122	1.22E-02	-1.51
Small Cajal body-specific RNA 1	SCARNA1	1.24E-02	-1.86
Small nucleolar RNA, H/ACA box 21	SNORA21	1.32E-02	-1.57
Golgin A6 family, member A	GOLGA6A	1.38E-02	1.72
Small nucleolar RNA, H/ACA box 52	SNORA52	1.40E-02	-1.64
Glutathione S-transferase alpha 3	GSTA3	1.42E-02	1.64
Small nucleolar RNA, H/ACA box 23	SNORA23	1.48E-02	-1.62
Small nucleolar RNA, H/ACA box 54	SNORA54	1.55E-02	-1.74

Refer to legend in Supplementary Table 2.

Supplementary Table 6: Effect of metformin on canonical pathways involved in CD34^{+} cells cultured in combined hyperglycaemia-hypoxia for 3 hours.

Canonical Pathways	*P*-value	Molecules
Mitochondrial Dysfunction	2.0E-03	↓COX6B1, ↓ATP5D, ↓COX8A, ↑MAPK9, ↓NDUFA3, ↓CYB5A, ↓COX5B, ↓MT-ND2
Triacylglycerol Biosynthesis	7.1E-03	↓GPAM, ↑AGPAT9, ↑ELOVL6
MAPK Signaling	7.9E-03	↓CXCL10, ↓MAP2K7, ↓PLA2G10, ↑MAPK9
Type 1 Diabetes Mellitus Signaling	9.5E-03	↓SOCS1, ↓MAP2K7, ↑IKBKG, ↓FCER1G, ↑MAPK9
IL-8 Signaling	2.3E-02	↑IKBKG, ↓ICAM1, ↓RHOG, ↓VEGFB, ↑MAPK9, ↑IRAK4

The arrow ↑ indicates gene is upregulated and ↓ indicates gene is downregulated. Key: *AGPAT9*: 1-acylglycerol-3-phosphate O-acyltransferase 9; *ATP5D*: ATP synthase, H+ transporting, mitochondrial F1 complex, delta subunit; *COX5B*: cytochrome c oxidase subunit Vb; *COX6B1*: cytochrome c oxidase subunit VIb polypeptide 1 (ubiquitous); *COX8A*: cytochrome c oxidase subunit VIIIA (ubiquitous); *CXCL10*: chemokine (C-X-C motif) ligand 10; *CYB5A*: cytochrome b5 type A (microsomal); *ELOVL6*: ELOVL Fatty Acid Elongase 6; *FCER1G*: Fc fragment of IgE, high affinity I, receptor for; gamma polypeptide; *GPAM*: glycerol-3-phosphate acyltransferase, mitochondrial; *ICAM1*: intercellular adhesion molecule 1, *IKBKG*: inhibitor of Kappa light polypeptide gene enhancer in B-Cells, kinase gamma; *IRAK4*: interleukin-1 receptor-associated kinase 4; *MAPK9*: mitogen-activated protein kinase 9; *MAP2K7*: mitogen-activated protein kinase 7; *MT-ND2*: mitochondrially Encoded NADH Dehydrogenase 2; *NDUFA3*: NADH dehydrogenase (ubiquinone) 1 alpha subcomplex, 3, 9kDa; *PLA2G10:* phospholipase A2, group X; *RHOG*: ras homolog gene family, member G (rho G); *SOCS1*: suppressor of cytokine signaling 1; *VEGFB*: vascular endothelial growth factor B.

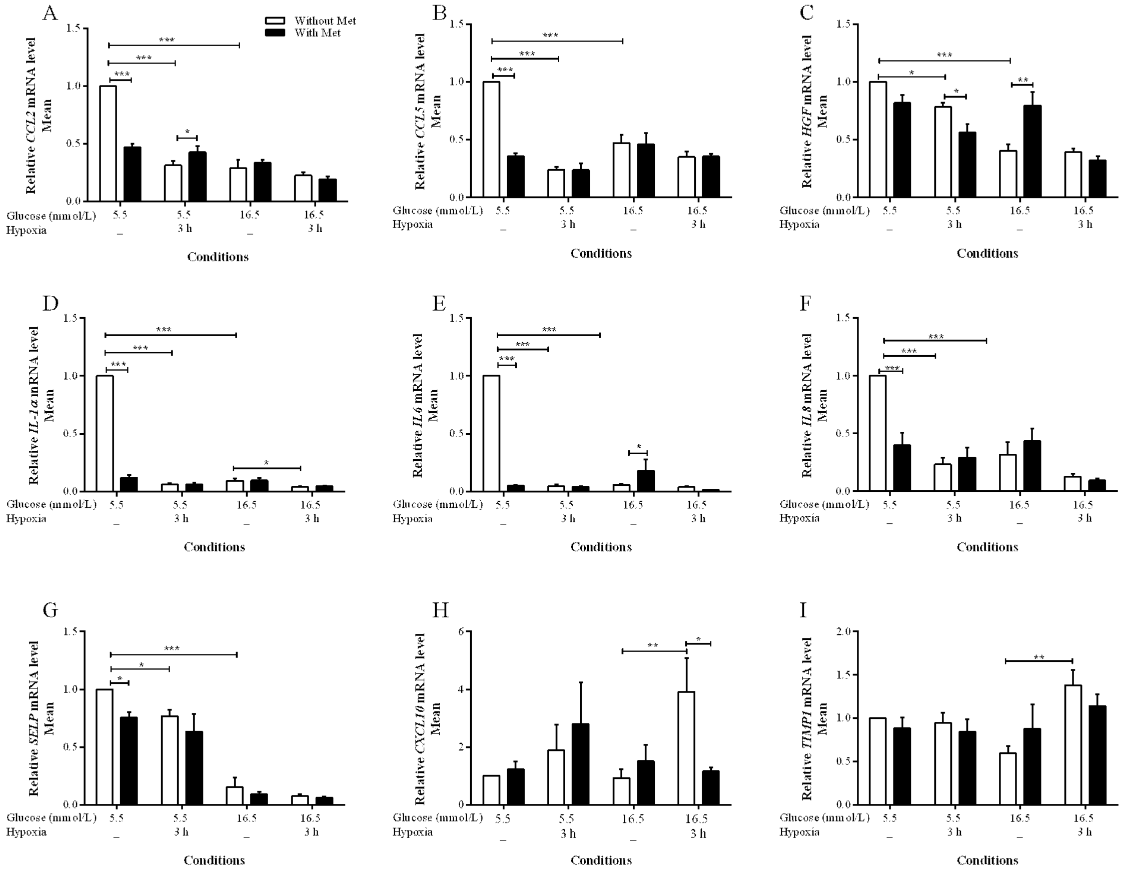

Supplementary Figure 3: Validation of selected pro-angiogenic factors and angiogenic inhibitors in CD34$^+$ cells by qRT-PCR. The variation in mRNA levels of (A) *CCL2,* (B) *CCL5,* (C) *HGF,* (D) *IL-1a,* (E) *IL-6,* (F) *IL-8,* (G) *SELP* (H) *CXCL10* and (I) *TIMP1* was measured by qRT-PCR (n = 3). Results are presented as ± SEM and were statistically analysed using one-way ANOVA followed by Fisher's LSD test. Data for effects of hypoxia and hyperglycemia were compared with control (5.5 mmol/L), data for the effect of hyperglycemia-hypoxia were compared with hyperglycemia. Whereas, data for cells treated with metformin were compared with the corresponding metformin-untreated condition. $^{*}P < 0.05$, $^{**}P < 0.01$, $^{***}P < 0.001$.

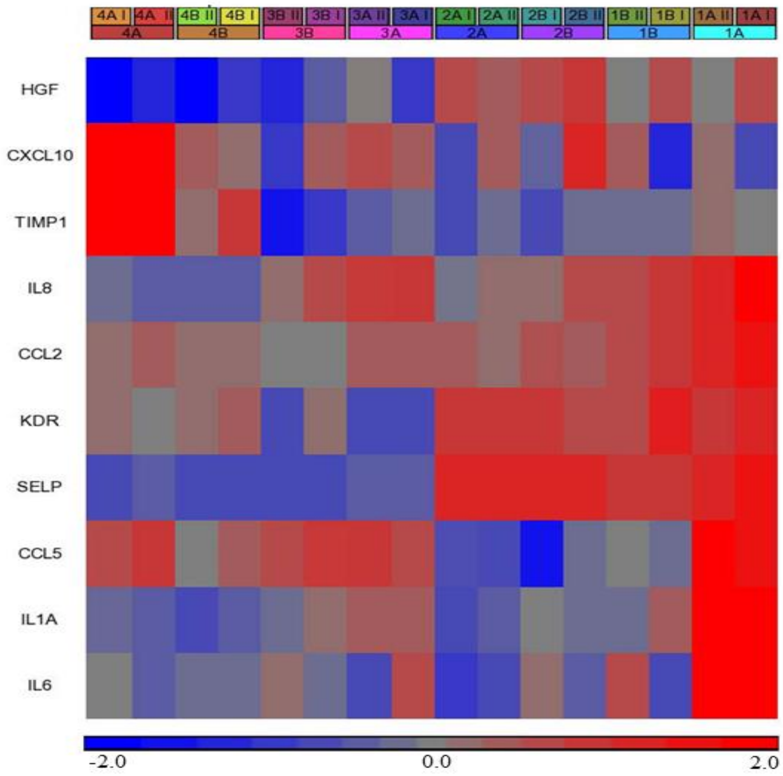

Supplementary Figure 4: Heatmap of the pro-angiogenic and angiogenic inhibitors in CD34$^+$ stem cells. Results were generated by transcriptomic analysis of 10 different experimental conditions each processed in two technical replicates resulting in 20 array experiments using Affymetrix microarray after exposure of CD34$^+$ cells to 4% hypoxia for 3 hours either under euglycaemia or hyperglycaemia in the presence or absence of metformin. The heatmap was generated by using Partek software. Red shading indicates upregulated genes, whereas blue shading indicates downregulated genes, and grey shading indicates non-affected genes. Key: 1A: euglycaemia, 1B: euglycaemia with metformin, 2A: euglycaemia with hypoxia, 2B: euglycaemia with hypoxia + metformin, 3A: hyperglycaemia, 3B: hyperglycaemia with metformin, 4A: hyperglycaemia with hypoxia, 4B: hyperglycaemia with hypoxia + metformin.

Supplementary Table 7: Comparison of microarray and real-time PCR data on CD34+ cells treated with hypoxia, hyperglycaemia and hyperglycaemia-hypoxia in the presence and absence of metformin.

Condition	Euglycaemia + metformin versus euglycaemia				Euglycaemia-hypoxia versus euglycaemia				Euglycaemia-hypoxia + metformin versus euglycaemia + hypoxia				Hyperglycaemia versus euglycaemia				Hyperglycaemia + metformin versus hyperglycaemia				Hyperglycaemia-hypoxia versus hyperglycaemia				Hyperglycaemia-hypoxia + metformin versus hyperglycaemia-hypoxia			
**Gene**	Microarray		qRT-PCR		Microarray		qRT-PCR		Microarray		qRT-PCR		Microarray		qRT-PCR		Microarray		qRT-PCR		Microarray		qRT-PCR		Microarray		qRT-PCR	
	FC	*P*	FC	*P*	FC	*P*	FC	*P*	FC	*P*	FC	*P*	FC	*P*	FC	*P*	FC	*P*	FC	*P*	FC	*P*	FC	*P*	FC	*P*	FC	*P*
**CCL2**	-1.7	3.2E-03	-2.1	< 0.001	-2.3	1.3E-04	-3.2	< 0.001	1.2	1.4E-01	1.3	3.7E-02	-2.1	2.5E-04	-3.4	< 0.001	-1.2	1.5E-01	1.2	3.6E-01	-1.1	5.7E-01	-1.2	2.3E-01	-1.3	6.9E-02	-1.2	5.2E-01
**CCL5**	-1.8	4.7E-04	-2.8	< 0.001	-2.3	3.1E-05	-4.2	< 0.001	1.0	8.2E-01	1.0	9.6E-01	-1.5	5.8E-03	-2.1	< 0.001	-1.0	7.6E-01	-1.0	8.3E-01	-1.0	8.4E-01	-1.3	8.5E-02	-1.2	2.5E-01	1.0	9.4E-01
**HGF**	-1.1	7.2E-01	1.2	8.6E-02	1.0	8.9E-01	-1.3	4.5E-02	-1.0	8.9E-01	-1.3	3.9E-02	-1.3	3.7E-02	-2.5	< 0.001	-1.2	1.5E-01	1.7	1.0E-03	-1.6	2.3E-03	1.0	9.0E-01	1.1	4.2E-01	-1.3	4.7E-01
**IL-1α**	-3.7	2.4E-05	-8.3	< 0.001	-4.7	5.2E-06	-16.7	< 0.001	1.1	6.5E-01	1.0	1.0E+00	-3.8	1.9E-05	-11.1	< 0.001	-1.2	3.1E-01	1.0	9.2E-01	-1.5	5.0E-02	-25.0	1.4E-02	1.1	7.6E-01	1.0	8.7E-01
**IL-6**	-2.2	1.7E-04	-20.0	< 0.001	-2.5	5.5E-05	-20.0	< 0.001	-1.0	9.6E-01	-1.1	9.1E-01	-2.1	2.5E-04	-16.7	< 0.001	1.1	7.5E-01	2.8	1.4E-02	-1.1	6.2E-01	-1.3	6.8E-01	1.0	8.4E-01	-2.8	6.1E-01
**IL-8**	-1.6	1.1E-02	-2.5	< 0.001	-2.4	9.5E-05	-4.3	< 0.001	1.2	1.8E-01	1.2	5.5E-01	-1.7	4.7E-03	-3.1	< 0.001	-1.3	1.5E-01	1.3	2.5E-01	-2.1	3.5E-04	-2.4	6.1E-02	-1.3	1.4E-02	-1.3	7.3E-01
**SELP**	-1.4	4.2E-03	-1.3	2.5E-02	-1.2	1.3E-01	-1.3	3.2E-02	1.1	2.5E-01	-1.1	2.1E-01	-5.0	3.3E-09	-6.7	< 0.001	-1.1	1.5E-01	-1.1	5.5E-01	-1.1	4.9E-01	-1.3	4.6E-01	1.0	7.5E-01	-1.1	8.8E-01
**CXCL10**	1.1	6.4E-01	1.2	8.2E-01	1.2	5.4E-01	1.9	3.7E-01	1.4	2.1E-01	1.4	3.7E-01	1.6	6.9E-02	-1.1	9.4E-01	-1.4	1.7E-01	1.6	5.6E-01	1.9	2.0E-02	3.9	7.0E-03	-2.0	1.3E-02	-3.3	1.1E-02
**TIMP1**	1.2	7.4E-02	-1.1	5.7E-01	1.1	2.1E-01	-1.0	7.9E-01	-1.0	7.8E-01	-1.1	6.2E-01	1.1	5.1E-01	-1.6	5.6E-02	-1.2	1.6E-01	1.5	1.7E-01	2.4	6.3E-06	1.4	1.0E-03	-1.7	3.9E-04	-1.3	2.5E-01

The highlighted data showed non-concordance in gene expression between qRT-PCR and microarray. Although hyperglycaemia and metformin resulted in an increase of mRNA levels of *HGF* and *IL-6* this remained below normal level observed at euglycaemia.
